# Supplementary material for: High protein copy number is required to suppress stochasticity in the cyanobacterial circadian clock
Source: Nat Commun. 2018 Aug 1;9:3004. doi: 10.1038/s41467-018-05109-4 (PMC6070526; doi:10.1038/s41467-018-05109-4)
Supplement: Supplementary file 1 — Supplementary Information [file 41467_2018_5109_MOESM1_ESM.pdf]

High protein copy number is required to suppress stochasticity in the cyanobacterial circadian clock

Chew et al.

Supplementary Information

Supplementary Methods

<u>Identification of appropriate genomic promoters to drive *kaiB* and *kaiC* transcription</u>

A major design criterion for the copy number tunable strain is to be able to express the Kai proteins in an expression range that spans from far below wild type levels up through wild type levels or above. The synthetic riboswitch has been shown previously at maximal induction to express genes at only ~25% of the expression levels obtained without the riboswitch[1], necessitating the use of a promoter that is stronger than the native *kaiBC* promoter. Additionally, for this study, we wished to focus our investigation of the clock molecular noise to the post-translational oscillator; thus, we aimed to choose promoters that were previously reported to lack circadian transcriptional-translational feedback, which contributes to clock period robustness[2].

We identified candidate promoters that met these criteria by combining global transcriptomic datasets from[3] and[4], and we examined genes whose mRNA was expressed from 4-40 times the expression level of *kaiBC* mRNA and that were also arrhythmic. To identify promoter sequences for these genes, we examined regions 500-750 bp upstream of their 5'-UTR, taking care to consider the 5'-UTR of the upstream-most gene if the transcript encoded a multi-gene operon. Only taking the sequence upstream of the 5'-UTR ensured that any untranslated regions would have minimal effect on riboswitch folding and function.

As a result of this analysis, we identified six candidate promoters from the following genes: Synpcc7942_0065, Synpcc7942_0089, Synpcc7942_1152, Synpcc7942_0321, Synpcc7942_0416, and Synpcc7942_1048. To test which of these promoters expressed Kai protein at the appropriate levels, we created strains of *Synechococcus* where *kaiA* expression is controlled by its native promoter but *kaiB* and *kaiC* expression is controlled by both the candidate promoter and the synthetic riboswitch (in the bioluminescent circadian reporter background). We successfully obtained clones carrying promoters Synpcc7942_0321, Synpcc7942_0416, and Synpcc7942_0089, and we compared the expression levels of KaiB and KaiC in these strains to wild type levels at either 0 μM theophylline or 2 mM theophylline by western blot. From this blot (data not shown), we identified Synpcc7942_0321 (*PglnB*) as a promoter of the appropriate strength.

<u>Identifying functionally equivalent light levels across experimental setups</u>

Due to the metabolic impact of light levels on overall protein expression in cyanobacteria (and therefore Kai copy number), we designed the experiments to ensure that cells were exposed to functionally equivalent light levels between the setups used to incubate cells for TopCount bioluminescence, western blot, and microscope experiments. Absolutely equal overhead illumination between conditions does not necessarily result in functionally equivalent light levels due to physical differences in experimental apparatus, such as the absorbance of light by the walls in the black 96-well plates, or the placement of BG11 agar above cells on the microscope. Additional differences may result from whether the cells are incubated in liquid media (western blots) or on top of/underneath solid media (TopCount/microscope experiments, respectively).

To identify functionally equivalent light levels, we incubated cells in the appropriate experimental setups for TopCount, western blot, and microscope experiments as described in the methods above. For the TopCount and western blot setups (powered by the Arduino-controlled LEDs), we exposed cells expressing YFP under control of the IPTG-inducible *trc* promoter (MRC1036) to varying levels of light while simultaneously incubating cells on the microscope at a fixed reference light level. For all experimental setups, cells were maximally induced with 1

mM IPTG. After 48 hours of incubation, cells were washed off the agar surface (for TopCount experiments) or taken directly from liquid culture (for western blot experiments) and placed on the microscope and imaged alongside cells grown on the microscope. The average single cell intensity under each condition was quantified with the CellProfiler software suite[5], and we determined light intensities for the TopCount and western blot setups that gave rise to cell fluorescence that matched cells grown on the microscope. The functionally equivalent light intensities were ~2 μmol photons m^{-2} s^{-1} for cells on the microscope, ~8.8 μmol photons m^{-2} s^{-1} for cells in liquid culture in 96-well black plates (e.g. Western blot cultures), and ~20 μmol photons m^{-2} s^{-1} for cells on solid media in 96-well black plates (e.g. TopCount experiments). These light intensities were used for all subsequent experiments.

Details of model implementation

The mathematical model we use here (Supplementary Fig. 5) is a simplified model which treats some elements of the biochemistry of the system abstractly, but captures known aspects of the Kai system that can produce oscillations. It has similarities to previously published models, where the functional units are KaiC hexamers[6,7]. First, the sequence of phosphorylation and dephosphorylation of KaiC occurs in an ordered fashion, reflected in an initial phase where phosphorylation occurs (yellow box in Supplementary Fig. 5, analogous to Thr432 phosphorylation dominant) until a threshold level of peak phosphorylation, after which a dephosphorylation phase initiates (blue box in Supplementary Fig. 5, analogous to Ser431 phosphorylation dominant). The ordering of the phosphorylation sites prevents the system from crossing between the intermediate states in the yellow and blue boxes. Second, KaiC only phosphorylates when bound to KaiA and otherwise dephosphorylates. This results in effective phosphorylation rates that depend on the concentration of KaiA, as observed experimentally[8]. Third, KaiC enters the dephosphorylation phase (blue box), is coincident with adopting a state that is bound to KaiB (likely ADP bound in the N-terminal domain of KaiC)[9]. These KaiBC complexes can bind and sequester KaiA. We assume that binding of KaiB is highly cooperative, so that 6 KaiB monomers bind to a single KaiC hexamer, and that each of these KaiB monomers is capable of inhibiting one KaiA dimer and does so with very high affinity so that KaiA is not released until that KaiC hexamer exits the dephosphorylation phase[10]. Additionally, we assume KaiB is present in excess and that KaiBC complex formation does not significantly deplete cellular KaiB, and thus we do not explicitly account for KaiB amounts in the model.

We designed the model to be flexible so that the number of reaction steps in the phosphorylation cycle m can be varied. Each elementary reaction that changes the state of a KaiC hexamer occurs with a rate constant k which can then be rescaled so that the oscillatory period remains independent of m. For a given value of m, the deterministic model is defined by the ordinary differential equations below.

$[C]$	Concentration of KaiC hexamers during phosphorylation cycle that are unbound to KaiA
$[\mathrm{AC}]$	Concentration of KaiC hexamers during phosphorylation cycle that are bound to KaiA
$[\mathrm{BC}]$	Concentration of KaiC hexamers during dephosphorylation cycle that are bound to KaiB and unbound to KaiA

[ABC]	Concentration of KaiC hexamers during dephosphorylation cycle bound to KaiB and sequestering KaiA
$[A]$	Concentration of KaiA dimers with free, unbound KaiA denoted as $[A_{\text{free}}]$ and total amount of initial KaiA denoted as $[A_{\text{tot}}]$

A given KaiC hexamer can exist in varying states of phosphorylation, from a minimum of 0 to a maximum of m. Here, i represents phosphorylation state during the phosphorylation cycle and j represents phosphorylation state during the dephosphorylation cycle, and we first consider cases where $0 < i < m-1$ and $0 < j < m$:

$$\frac{d[C_i]}{dt} = k_{\text{dephos}} \cdot ([C_{i+1}] - [C_i]) + k_{A_{\text{off}}} \cdot [\text{AC}_i] - k_{A_{\text{on}}} \cdot [C_i] \cdot [A_{\text{free}}] \tag{1}$$

$$\frac{d[\text{AC}_i]}{dt} = k_{\text{phos}} \cdot ([\text{AC}_{i-1}] - [\text{AC}_i]) + k_{A_{\text{on}}} \cdot [C_i] \cdot [A_{\text{free}}] - k_{A_{\text{off}}} \cdot [\text{AC}_{\text{i}}] \tag{2}$$

$$\frac{d[\text{BC}_j]}{dt} = k_{\text{dephos}} \cdot ([\text{BC}_{j+1}] - [\text{BC}_j]) - k_{\text{ABC}} \cdot [\text{BC}_j] \tag{3}$$

$$\frac{d[\text{ABC}_j]}{dt} = k_{\text{dephos}} \cdot \left([\text{ABC}_{j+1}] - [\text{ABC}_j]\right) + k_{\text{ABC}} \cdot [\text{BC}_j] \cdot [A_{\text{free}}] \tag{4}$$

$$[A_{\text{free}}] = \max\left(0, [A_{\text{tot}}] - \sum_{i=0}^{m-1} [\text{AC}_i] - 6 \sum_{j=1}^{m} \left[\text{ABC}_j\right]\right) \tag{5}$$

For special cases $i = 0, m-1$:

$$\frac{d[C_0]}{dt} = k_{\text{dephos}} \cdot ([C_1] + [\text{BC}_1] + [\text{ABC}_1]) + [C_1](k_{A_{\text{off}}} - k_{A_{\text{on}}}) \tag{6}$$

$$\frac{d[\text{AC}_0]}{dt} = -k_{\text{phos}} \cdot [\text{AC}_0] + k_{A_{\text{on}}} \cdot [C_0] \cdot [A_{\text{free}}] - k_{A_{\text{off}}} \cdot [\text{AC}_0] \tag{7}$$

$$\frac{d[C_{m-1}]}{dt} = k_{\text{dephos}} \cdot ([\text{BC}_m] - [C_{m-1}]) + k_{A_{\text{off}}} \cdot [\text{AC}_{m-1}] - k_{A_{\text{on}}} \cdot [C_{m-1}] \cdot [A_{\text{free}}] \tag{8}$$

$$\frac{d[\text{AC}_{m-1}]}{dt} = k_{\text{phos}} \cdot ([\text{AC}_{m-2}] - [\text{AC}_{m-1}]) + k_{A_{\text{on}}} \cdot [C_{m-1}] \cdot [A_{\text{free}}] - k_{A_{\text{off}}} \cdot [\text{AC}_{m-1}] \tag{9}$$

And for $j = m$:

$$\frac{d[\mathrm{BC}_m]}{dt} = k_{\mathrm{phos}} \cdot [\mathrm{AC}_{m-1}] - 2 \cdot k_{\mathrm{dephos}} \cdot [\mathrm{BC}_m] - k_{\mathrm{ABC}} \cdot [\mathrm{BC}_m] \tag{10}$$

$$\frac{d[\mathrm{ABC}_m]}{dt} = -k_{\mathrm{dephos}} \cdot [\mathrm{ABC}_m] + k_{\mathrm{ABC}} \cdot [\mathrm{BC}_m] \cdot [A_{\mathrm{free}}] \tag{11}$$

To interpolate between full strength negative feedback (*Synechococcus*-like) and no negative feedback (*Prochlorococcus*-like), we make the following modifications to the model. First, we introduce an additional state of KaiC in the phosphorylating cycle, C^*, which can phosphorylate in a KaiA-independent manner, consistent with experimental evidence indicating that Prochlorococcus KaiC can autophosphorylate in the absence of KaiA[11]. KaiC can thus transition to one of two states that promote phosphorylation: one in which it binds to KaiA (AC), and one in which it does not (C^*). The relative probability of these states is determined by a parameter η which can take values between 0 and 1. When $\eta = 1$, all phosphorylation is KaiA-dependent (the Synechococcus-like model). When $\eta = 0$, all phosphorylation occurs via the KaiA-independent state. Lastly, we introduce environmental input in the form of the cellular ATP/(ATP + ADP) ratio, which has been shown to alter KaiC phosphorylation in *Synechococcus*, leading to entrainment to metabolic signals[12]. Here, we model this effect by allowing the ATP/ADP ratio to influence the rate at which KaiC enters a phosphorylation-competent state, and we denote the fraction of total (ATP + ADP) that is ATP as f_{ATP}. This hypothetical mechanism allows phosphorylation of *Prochlorococcus* KaiC to depend on the light-dark cycle through metabolism.

Specifically, the rate at which KaiC enters the KaiA-bound phosphorylating state changes:

$$k_{A_{\mathrm{on}}} \rightarrow k_{A_{\mathrm{on}}} \cdot \eta \cdot f_{\mathrm{ATP}}$$

Similarly, the total rate at which KaiC switches to the KaiA-independent phosphorylating state can be expressed as:

$$k_{C \rightarrow C^*} \cdot (1 - \eta) \cdot f_{\mathrm{ATP}}$$

In total, the entire modified system of equations can be written, modifying equations (1) and (2) to produce equations (12) and (13). First, we consider cases where $0 < i < m - 1$ (the reactions on the dephosphorylating cycle remain unchanged):

$$\frac{d[C_i]}{dt} = k_{\mathrm{dephos}} \cdot ([C_{i+1}] - [C_i]) + k_{A_{\mathrm{off}}} \cdot [\mathrm{AC}_i] - k_{A_{\mathrm{on}}} \cdot \eta \cdot f_{\mathrm{ATP}} \cdot [C_i] \cdot [A_{\mathrm{free}}] - k_{C \rightarrow C^*} \cdot (1 - \eta) \cdot f_{\mathrm{ATP}} \cdot [C_i] \tag{12}$$

$$\frac{d[\mathrm{AC}_i]}{dt} = k_{\mathrm{phos}} \cdot ([\mathrm{AC}_{i-1}] - [\mathrm{AC}_i]) + k_{A_{\mathrm{on}}} \cdot \eta \cdot f_{\mathrm{ATP}} \cdot [C_i] \cdot [A_{\mathrm{free}}] - k_{A_{\mathrm{off}}} \cdot [\mathrm{AC}_i] \tag{13}$$

$$\frac{d[C^*{}_i]}{dt} = k_{\text{phos}} \cdot ([C^*{}_{i-1}] - [C^*{}_i]) + k_{C\to C^*} \cdot (1-\eta) \cdot f_{\text{ATP}} \cdot [C_i] - k_{C^*\to C} \cdot [C^*{}_i] \tag{14}$$

Equations (6)-(9) are rewritten to produce equations (15)-(18) for $i = 0, m-1$:

$$\frac{d[C_0]}{dt} = k_{\text{dephos}} \cdot ([C_1] + [\text{BC}_1] + [\text{ABC}_1]) + k_{A_{\text{off}}} \cdot [\text{AC}_0] - k_{A_{\text{on}}} \cdot \eta \cdot f_{\text{ATP}} \cdot [C_0] \cdot [A_{\text{free}}] + k_{C\to C^*} \cdot (1-\eta) \cdot f_{\text{ATP}} \cdot [C_0] \tag{15}$$

$$\frac{d[\text{AC}_0]}{dt} = -k_{\text{phos}} \cdot [\text{AC}_0] + k_{A_{\text{on}}} \cdot \eta \cdot f_{\text{ATP}} \cdot [C_0] \cdot [A_{\text{free}}] - k_{A_{\text{off}}} \cdot [\text{AC}_0] \tag{16}$$

$$\frac{d[C_{m-1}]}{dt} = k_{\text{dephos}} \cdot ([\text{BC}_m] - [C_{m-1}]) + k_{A_{\text{off}}} \cdot [\text{AC}_{m-1}] - k_{A_{\text{on}}} \cdot \eta \cdot f_{\text{ATP}} \cdot [C_{m-1}] \cdot [A_{\text{free}}] + k_{C\to C^*} \cdot (1-\eta) \cdot f_{\text{ATP}} \cdot [C_{m-1}] \tag{17}$$

$$\frac{d[\text{AC}_{m-1}]}{dt} = k_{\text{phos}} \cdot ([\text{AC}_{m-2}] - [\text{AC}_{m-1}]) + k_{A_{\text{on}}} \cdot \eta \cdot f_{\text{ATP}} \cdot [C_{m-1}] \cdot [A_{\text{free}}] - k_{A_{\text{off}}} \cdot [\text{AC}_{m-1}] \tag{18}$$

Deterministic simulations of the system were implemented by using fourth-order Runge-Kutta numerical integration with a step size of 0.01 hours.

To simulate the system stochastically, we reimplemented the reactions shown above using the Gillespie algorithm[13], denoting the copy number of a particular species as N_{species}. Thus, the rate at which KaiC binds to KaiA during the phosphorylation cycle can be rewritten:

$$k_{A_{\text{on}}} \cdot \eta \cdot f_{\text{ATP}} \cdot N_{C_i} \cdot N_{A_{\text{free}}} \rightarrow \; k_{A_{\text{on}}} \cdot \eta \cdot f_{\text{ATP}} \cdot N_{C_i} \cdot [A_{\text{tot}}] \cdot \frac{N_{A_{\text{free}}}}{N_{A_{\text{tot}}}}$$

And the rate at which KaiBC binds to KaiA to sequester it during the dephosphorylation cycle can be rewritten:

$$k_{\text{ABC}} \cdot N_{\text{BC}_j} \cdot N_{A_{\text{free}}} \rightarrow k_{\text{ABC}} \cdot N_{\text{BC}_j} \cdot [A_{\text{tot}}] \cdot \frac{N_{A_{\text{free}}}}{N_{A_{\text{tot}}}}$$

In experiments, changing theophylline concentrations changes copy number but also changes concentration of Kai proteins. To reflect this in the stochastic implementation where the cell volume remains constant over varying copy numbers, the concentration $[A_{\text{tot}}]$ is scaled by the overall Kai copy number relative to wild type, where wild type copy number of KaiC is specified to be 1200 hexamers (derived from experiments in this study measuring Kai copy number to be ~8000).

A table of simulation parameter values for this study are listed in Supplementary Table 1. Rate constants in the model were selected such that their relative magnitudes were reasonably consistent with previous findings (e.g. the KaiA binding rate is faster than the disassociation rate, and important slow steps in the cycle are reactions within a KaiC hexamer: phosphorylation, dephosphorylation, and switching into a KaiB-binding competent state[14]). Values for KaiA-KaiC stoichiometries for stimulating phosphorylation (1:1 stoichiometry) and for sequestration (6:1 stoichiometry) were chosen based on reported literature values[10,15]. As a final step, all model rate constants were scaled proportionally such that the period of oscillations was 24 hours.

Determining model timing error

To quantify the amount of oscillatory noise in the model at varying copy numbers (as shown in Fig. 3), the simulation was first initiated with all KaiC completely dephosphorylated and unbound to KaiA (C_0). From this initial condition, the simulation was simulated deterministically, and the system quickly converged to a limit cycle. To allow the system to reach steady state oscillations, the simulation was run for 1000 hours. The relative amounts of each KaiC species was then recorded and saved at the KaiC phosphorylation trough most immediately before the 1000 hour simulation endpoint. KaiC phosphorylation was calculated by summing up the total number of phosphorylated sites on all KaiC molecules in the simulation.

The save point was then used as initial conditions for all subsequent simulations, and concentrations of Kai proteins were converted to absolute copy number assuming a conversion factor of 1200 molecules$/1.56\ \mu M$. To more closely mimic the experimental data in which single cells grow and divide, the simulation was first run for 24 hours (the "mother cell"), at which point the simulation state was cloned into two simulations which were left to run independently thereafter (as the "daughter cells"). 24 hours later, the simulations were cloned and split again into four, and this was repeated for a total of 7 complete "generations", or 168 hours of simulation time for each individual simulation lineage. KaiC phosphorylation data over time was compiled for individual lineages, and to facilitate further downstream processing, the data were resampled at a time interval of 0.01 h with linear interpolation. Peak-to-peak measurements were then obtained in identical manner to that used for experimental data (outlined in the main text Methods) with the exception that a window size of 211 was used for the Savitsky-Golay smoothing filter on the model data before peak finding.

This simulation was repeated for varying copy numbers of Kai protein and for varying values of m as shown in Supplementary Fig. 6, with $m = 5$ providing the best fit to the experimental data as shown in Fig. 3D.

Noise sensitivity analysis of all molecular species in the model

We performed a noise sensitivity analysis to determine which molecular species in the model were most susceptible to noise. Specifically, we defined noise susceptibility as the average magnitude of phase shift induced by an instantaneous single perturbation of molecular noise in the amount of a given molecular species. Perturbations were simulated to be Poisson-like in nature in order to reflect the expected $1/\sqrt{N}$ noise scaling behavior, where N represents the copy number of a given species. Thus, we expected that molecular species that were present in smaller amounts should experience greater fluctuations in their amounts.

To precisely isolate the effect of a single perturbation of molecular noise, the model was first initialized from steady state oscillations (specifically after 1994.49 hours of deterministic simulation starting from dephosphorylated KaiC) and run deterministically for $24 + \Delta t$ hours,

where Δt represents a random interval of time picked from a uniform distribution between 0 and 24 hours. At this point a molecular noise perturbation was introduced by converting the concentration of a given molecular species to a discrete copy number (corresponding to 25% wild type copy number and using a conversion factor of 1200 molecules$/1.56\ \mu M$), at which point the copy number was resampled from a Poisson distribution whose mean was the initial copy number. This resampled copy number was converted back into a concentration value, and from here the simulation was run deterministically for an additional 48 hours.

The phase shift between the pre- and post-perturbation trajectories was calculated by taking the absolute value of the difference in the phases obtained from sinusoid fitting to both trajectories.

For each molecular species in the model, we repeated this process a total of n = 500 trials. We grouped molecular species into four categories: KaiAC complexes, unbound KaiC, KaiBC complexes, KaiABC complexes. For each category, we then calculated the average magnitude of phase shift, which is shown in Fig. 3E.

Determining the range of KaiABC complexes that switch KaiC between kinase and phosphatase modes

To calculate the dependence of KaiC net kinase rates on the number of KaiABC complexes present at a given moment in time, the average amount of each KaiC species was first calculated by averaging KaiC amounts over a 24 hour period sampled at 1 hour intervals. The 24 hour period over which individual KaiC amounts were recorded ranged from t = 1958.07 h (trough of phosphorylation) to t = 1982.07 in a deterministic simulation in which KaiC was initialized in the unphosphorylated state and unbound to KaiA. Once the average KaiC amounts were calculated, these amounts were used as the initial conditions for subsequent simulation.

Because the amount of free KaiA available to bind to KaiC depends on the amount of KaiABC complex, the simulation was allowed to reach an equilibrium of KaiA-KaiC binding by first allowing only the KaiA-KaiC binding/unbinding reactions to run for 20 hours, disabling any phosphorylation and dephosphorylation reactions. After KaiA-KaiC binding reached equilibrium, the net KaiC kinase rate was calculated by taking the sum of all rate constants for phosphorylating reactions and subtracting the sum of all rate constants for dephosphorylating reactions. This process was then repeated for varying amounts of KaiABC complexes.

The curve shown in Fig. 3F was produced by sampling the net kinase vs. KaiABC complex curve in discrete amounts corresponding to incrementing the copy number of KaiABC complexes by 1 and calculating the corresponding change in net kinase rate, and this was simulated for a KaiC copy number of 1,800.

Calculating clock/environment mutual information for varying feedback loop strengths

To determine the optimal feedback loop strength at varying Kai copy numbers, we varied the feedback loop strength in the presence of environmental fluctuations. Feedback loop strength was tuned by varying the parameter η to take values between 0 (no feedback loop) to 1 (full feedback strength). For each value of η, the simulation was first run deterministically for 50 cycles of 12h:12h light/dark cycles without environmental noise to reach steady state, initialized from unphosphorylated KaiC not bound to KaiA. Light/dark cycles were emulated by cycling ATP levels between 80% (day) and 40% (night), reflecting experimental values observed previously. These steady states were then used as the starting points for subsequent simulations.

For stochastic simulations, the simulations were run at constant concentration, where Kai copy number was changed by changing the effective simulation volume (thus cells with smaller Kai copy number will have proportionally smaller volumes). This reflects our experimental findings that while *Synechococcus* and *Prochlorococcus* have vastly different Kai copy numbers, the concentrations of Kai proteins are roughly equivalent in both organisms due to their difference in volume. The simulations were then run at varying copy numbers for 10 cycles of 12h:12h light/dark cycles with fluctuations.

Environmental fluctuations were simulated by adding fluctuations to a deterministic 12h:12h light/dark cycle as outlined above. In the case without external fluctuations, f_{ATP} is 80% during the day and 40% at night. When fluctuations are present, we instead generate f_{ATP} via a Markov process where the input signal switches to a new level with a mean waiting time of 1.33 hours. For each environmental transition, f_{ATP} is drawn from a normal distribution centered on 80% (day) or 40% (night) with either a standard deviation of 10% (intermediate environmental noise) or 25% (high environmental noise). Example plots of such ATP fluctuations are shown in Supplementary Fig. 10C. To facilitate downstream data processing, the data from each simulation was resampled at a rate of 0.01 hours using linear interpolation. For each copy number and value of η, the simulation was repeated a total of 900 times.

For mutual information calculations, the data from the last 5 cycles of 12h:12h light/dark was used. The multidimensional clock state was projected into 2 dimensions by quantifying the total amount of phosphorylation in the phosphorylating half of the cycle (P, yellow box in Supplementary Fig. 5) vs. the dephosphorylating half of the cycle (D, blue box in Supplementary Fig. 5).

Time data was binned into 24 bins, and phosphorylation data was binned into 100 x 100 bins. Mutual information (I) was calculated for each Kai copy number and value of η:

$$I(T, \langle P, D \rangle) = \sum_{t \in T} \sum_{\langle p,d \rangle \in \langle P,D \rangle} \mathrm{Pr}(t, \langle p, d \rangle) \cdot \log_2 \frac{\mathrm{Pr}(t, \langle p, d \rangle)}{\mathrm{Pr}(t)\mathrm{Pr}(\langle p, d \rangle)}$$

Here, T denotes the entire set of time points, and t denotes a specific time point. Similarly, $\langle P, D \rangle$ denotes the entire set of observed phosphorylation states, and $\langle p, d \rangle$ denotes a specific phosphorylation state observation. Pr denotes the probability function.

Determining the bifurcation point where oscillations lose stability as feedback strength decreases

The bifurcation point in Figures 4F-G (denoted by the horizontal dashed line) was determined for various values of feedback loop strength η and ATP/(ATP + ADP) ratios by the following method. For a given, constant ATP ratio and starting at $\eta = 1.0$, a deterministic simulation was run for 1000 hours, and a binary search was executed over varying values of η to determine the level of η where oscillations would disappear or become unstable. Oscillations were considered to be stable and present if the amplitude did not drop by more than 2% over the last 100 hours of the simulation and if the amplitude over the last 50 hours was greater than 10% of the oscillatory amplitude obtained at $\eta = 1.0$.When the input signal is constant, the bifurcation point depends on the ATP ratio value, so in the case of fluctuating environmental input, we defined the bifurcation point of the system to be the value of η above which oscillations were present and stable for all ATP ratio levels between 80% and 40%.

Supplementary Note 1

Estimate of the fraction of the total proteome occupied by Kai proteins for *Synechococcus*-like expression levels in *Prochlorococcus*

Given a cellular diameter of ~0.5 μm[16], an estimate for the cell volume of *Prochlorococcus* is ~0.1 fl assuming a spherical cell shape, a reasonable assumption based on electron micrographs (previous literature[17] and Supplementary Fig. 7). Cellular protein amount was measured to be ~15 fg/cell in *Prochlorococcus* (corresponding to ~150 mg/ml concentration), which was quantified by measuring the total protein yield from cell lysate by Bradford assay and dividing by the total number of cells in the sample as measured by flow cytometry. Protein concentration was estimated by dividing mg/ml by cell volume.

Given a protein amount of ~15 fg/cell and assuming an average protein molecular weight of 26.7 kDa in bacteria[18], we can estimate that there are ~10^5 copies of proteins/cell in *Prochlorococcus*. Wild type *Synechococcus* expresses a total of ~20,000 Kai proteins, which would constitute ~20% of the total proteome in *Prochlorococcus,* a large amount for an already minimal photosynthetic organism.

Supplementary Table 1
Parameters used in the Kai model.

Category	Parameter	Value	Description
Common parameters for all simulations	m	5 unless otherwise specified	Number of phosphorylation steps
	η	1 unless otherwise specified, varies from 0-1	Feedback loop strength
	f_{ATP}	1 unless otherwise specified, varies from 0-1	Environmental input
	dt	$0.01\ h^{-1}$	Step size for deterministic simulation
Rate constants	k_{phos}	$2m \cdot 0.04902\ h^{-1}$	Phosphorylation rate
	k_{dephos}	$2m \cdot 0.04902\ h^{-1}$	Dephosphorylation rate
	$k_{A_{\mathrm{on}}}$	$0.2451\ \mu M^{-1}\ h^{-1}$	Rate of KaiA/KaiC binding to switch KaiC to phosphorylating state
	$k_{A_{\mathrm{off}}}$	$0.02451\ h^{-1}$	Rate of KaiA/KaiC disassociation from phosphorylating state
	$k_{C \to C^*}$	$0.2451\ h^{-1}$	Rate of KaiC switching to KaiA-independent phosphorylating state
	$k_{C^* \to C}$	$0.02451\ h^{-1}$	Rate of KaiC switching from KaiA-independent phosphorylating state
	k_{ABC}	$110.80\ h^{-1}$	Rate of KaiC binding to KaiB to sequester KaiA
Deterministic simulation initial conditions	$[C_0]_{\mathrm{init}}$	$1.56\ \mu M$	Initial concentration of KaiC hexamers
	$[A_{\mathrm{tot}}]$	$2.0\ \mu M$	Initial concentration of KaiA dimers
	dt	$0.01\ h$	Step size for numerical integration
Stochastic simulation initial conditions	scale_factor	Varies	Scaling factor for copy number, where 1 indicates wild type-like levels
	$n_{C_{0_{\mathrm{init}}}}$	1200 · scale_factor	Initial copy number of KaiC hexamers
	$n_{A_{\mathrm{tot}}}$	1200 · scale_factor	Initial copy number of KaiA dimers

Supplementary Table 2

Calculated concentrations of Kai proteins in *S. elongatus* and *P. marinus* using western blotting copy number estimates and estimated cell volumes of 2 fl and 0.1 fl for *S. elongatus* and *P. marinus*, respectively.

	[KaiA]	[KaiB]	[KaiC]
Wild type *S. elongatus*	3.0 µM	9.0 µM	6.6 µM
P. marinus	N/A	n.d.	11.4 µM

Supplementary Figures

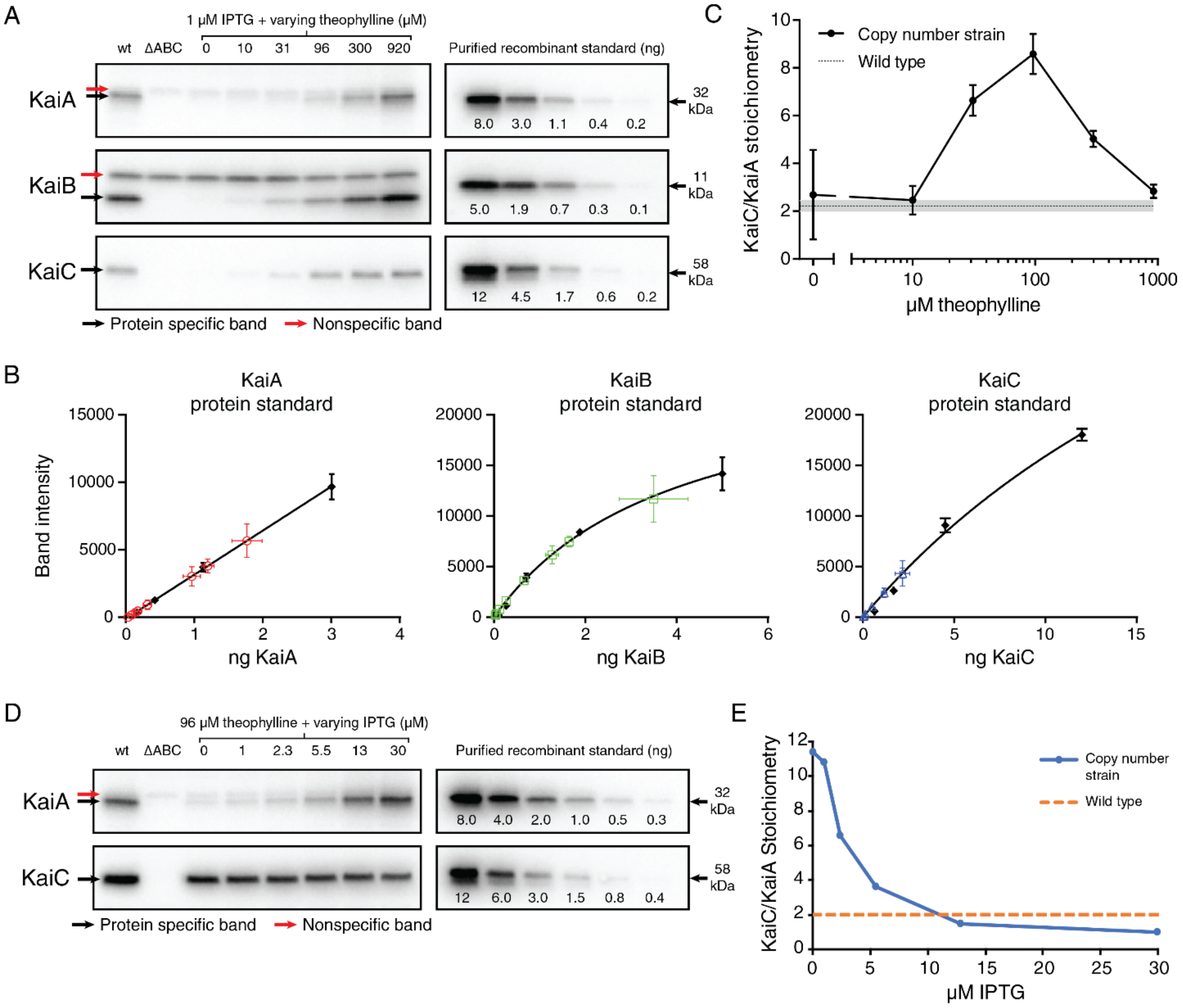

Supplementary Fig. 1

Characterization of copy number tunable strain. (**A**) Representative western blot images for data presented in Fig. 1C (n = 3 biological replicates). For each protein KaiA, KaiB, and KaiC, cell lysate and recombinant standards were blotted simultaneously on the same membrane. *Left*: cell lysate from wild type, *kaiABC* null, or copy number tunable cells incubated in 1 μM IPTG and various amounts of theophylline. For KaiA and KaiB western blots, 3 μg total cell lysate protein was loaded per well. For KaiC western blot, 0.75 μg total cell lysate protein was loaded per well. Protein specific bands are highlighted with black arrows, and nonspecific bands are highlighted with red arrows. *Right*: recombinant protein standards were all carried out in a dilution series, with the maximum and minimum amounts loaded per standard as follows: 8 ng to 0.16 ng for KaiA, 5 ng to 0.10 ng for KaiB, and 12 ng to 0.24 ng for KaiC. (**B**) Quantification of western blot protein standards (*black*) and cell lysate samples (*red, KaiA; green, KaiB; blue, KaiC*). Vertical error bars represent SEM of band intensity for both standards and samples, and horizontal error bars represent SEM of calculated protein abundance in cell lysate samples (n =

3). Solid curves show best fit to lines (KaiA) or rectangular hyperbolas (KaiB and KaiC). (**C**) Relative stoichiometry of KaiC to KaiA cellular copy number using data from (A). Error bars and shaded interval indicate the standard error of the mean. (**D**) *Left*: cell lysate from wild type, *kaiABC* null, or copy number tunable cells incubated in 96 μM theophylline and various amounts of IPTG. For both KaiA and KaiC, 3 μg total cell lysate protein was loaded per well. *Right*: recombinant protein standards were similarly carried out in a 2-fold dilution series, with the maximum and minimum amounts loaded per standard as follows: 8 ng to 0.25 ng for KaiA and 12 ng to 0.38 ng for KaiC. (**E**) Relative stoichiometry of KaiC to KaiA cellular copy number using data from (D).

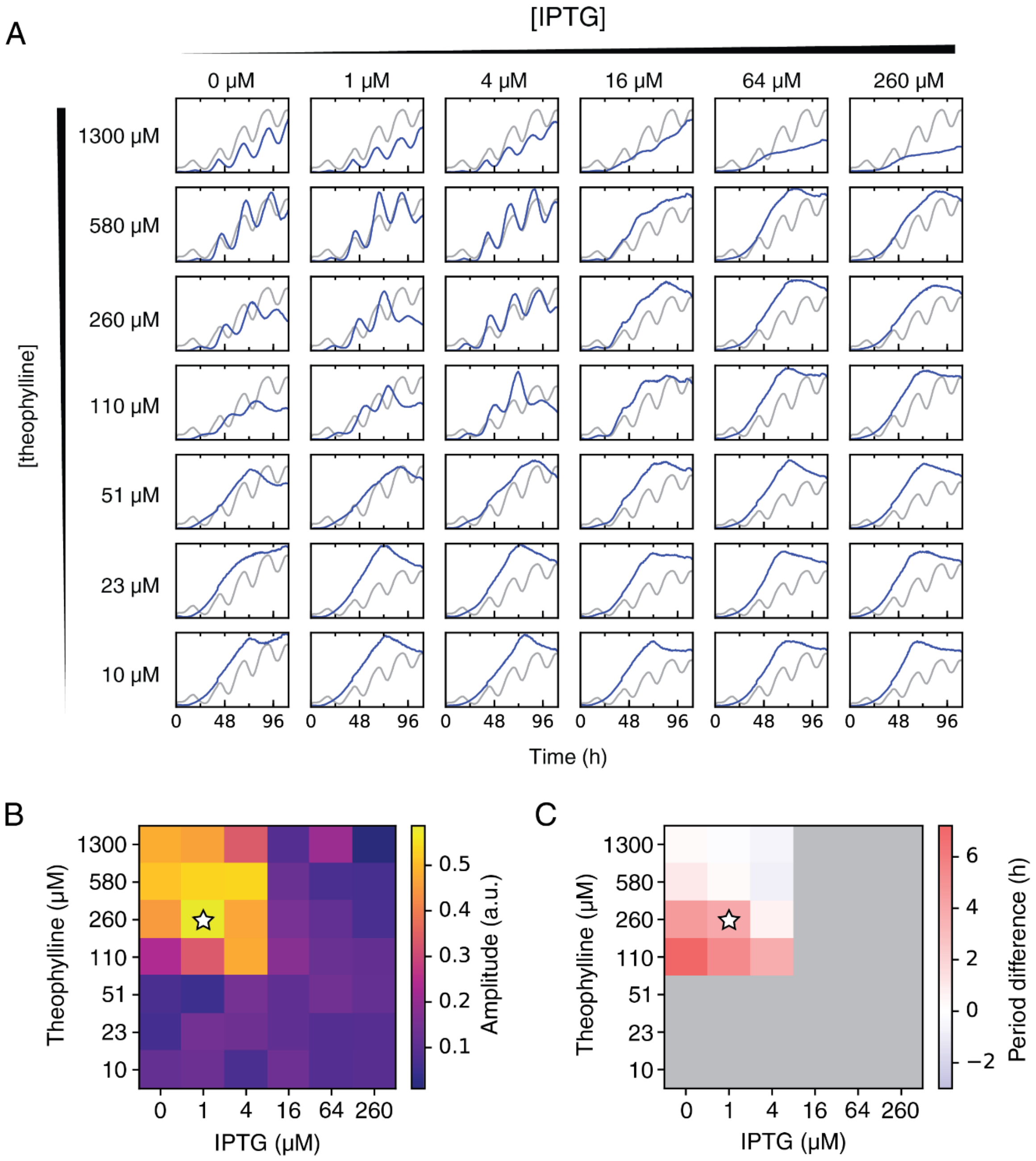

Supplementary Fig. 2

Bulk level oscillation data from the TopCount plate reader assay. (**A**) Bioluminescence traces over time in either wild type cells (*gray*) or copy number tunable cells plated on BG11-agar containing various amounts of inducer (*blue*). Shown are the averages of data from n = 12 individual wells for wild type and n = 4 individual wells for copy number tunable cells. Upward trend in bioluminescence is due to cell growth/division over the course of the experiment (the data shown are raw traces and are not normalized). (**B**) Heatmap of oscillation amplitude at various levels of inducer. Amplitudes are obtained from fitting data to a sinusoid after normalizing data for cell growth/division. White star indicates conditions that approximate wild type levels of Kai protein expression. (**C**) Heatmap of oscillation period relative to wild type

cells after similar fitting to sine curves after normalization. Gray areas indicate conditions where the oscillation amplitude was too weak to accurately determine period. White star indicates conditions that approximate wild type levels of Kai protein expression.

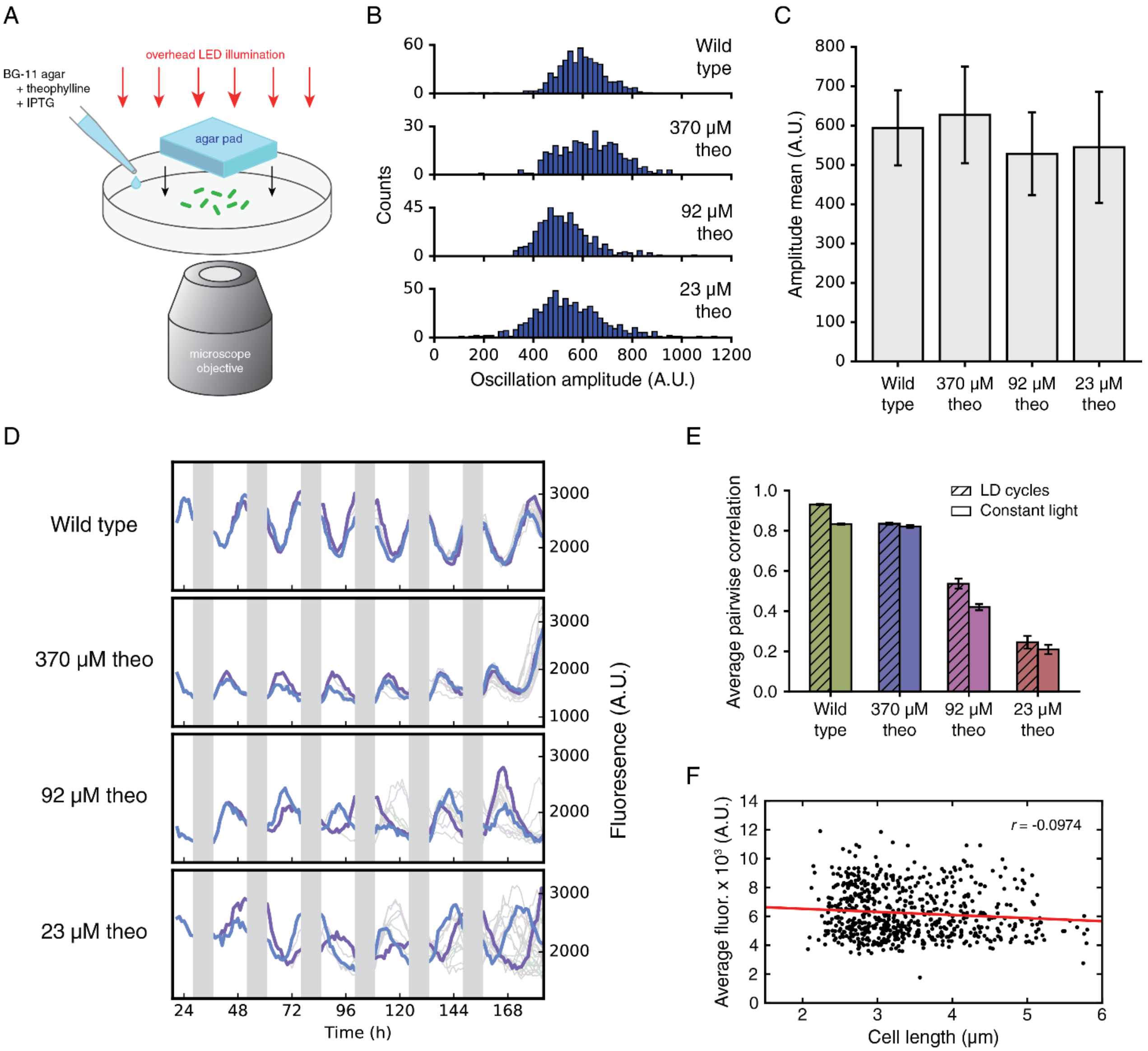

Supplementary Fig. 3

(**A**) Experimental setup for fluorescent time-lapse microscopy. Cells are incubated in 96-well plates (only a single well is shown, not drawn to scale) underneath an agar pad with uniform illumination. Additional BG11-agar containing inducers is added on top of the agar pad. The entire setup is contained within a light and temperature-controlled box held at 30°C. (**B-C**) Oscillation amplitude in wild type cells and in the copy number tunable strain at various theophylline concentrations and 1 μM IPTG, with histograms of oscillation amplitudes shown in (B) and quantification of mean oscillation amplitudes in (C) with error bars denoting standard deviations of amplitude distributions. (**D**) Time lapse fluorescence microscopy traces showing single cell oscillation trajectories (*gray*) with two representative lineages highlighted (*blue and purple*) in the presence of 16h:8h light-dark cycles followed by release into constant light. Periods of darkness (where images were not acquired) are indicated by gray rectangles. (**E**) Average pairwise Spearman correlations were calculated for all descendants within micro-colonies from t = 155-183 hours (release into constant light after LD cycles) or t = 127-155 hours

(constant light only, calculated from experimental data shown in Fig 2). Error bars indicate 95% bootstrapping confidence intervals (1000 iterations). Total numbers of correlations used for LD cycle data were n = 1313 (wild type), 1794 (370 μM), 1205 (92 μM), 1055 (23 μM), and for constant light, n = 10927 (wild type), 1663 (370 μM), 3673 (92 μM), 2354 (23 μM). (**F**) Constitutive YFP expression fluorescence as a function of cell length. Scatterplot of average integrated cell fluorescence in *S. elongatus* expressing YFP under control of the *trc* promoter with 1 mM IPTG, n = 659 cells. *r* indicates the Pearson correlation coefficient, and the red line indicates the best fit linear regression.

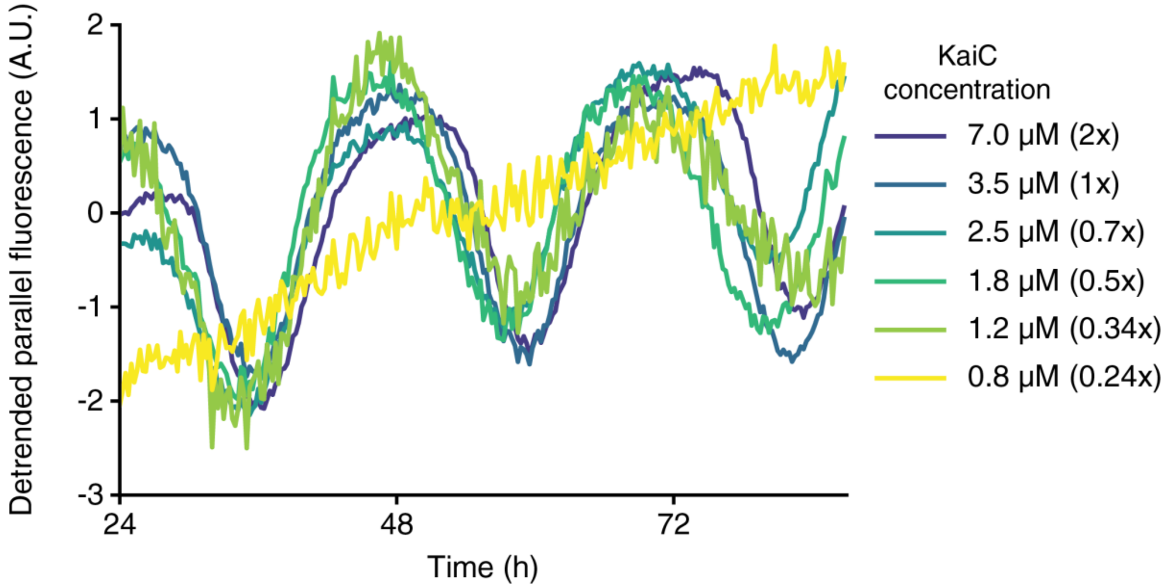

Supplementary Fig. 4

Parallel fluorescence of in vitro reactions containing fluorescein-conjugated KaiB (henceforth KaiB*) was measured over time. Across the reactions, the relative stoichiometry between all Kai proteins was held constant while the absolute Kai protein concentrations were multiples of the standard concentration, denoted here as “1x” and containing 1.5 uM KaiA, 3.5 uM KaiB, 3.5 uM KaiC, and 0.2 uM KaiB*. KaiC concentrations are listed in the legend as reference. Oscillatory signals were detrended using background fluorescence from a KaiB*-only control and were normalized such that mean signal = 0 and standard deviation = 1.

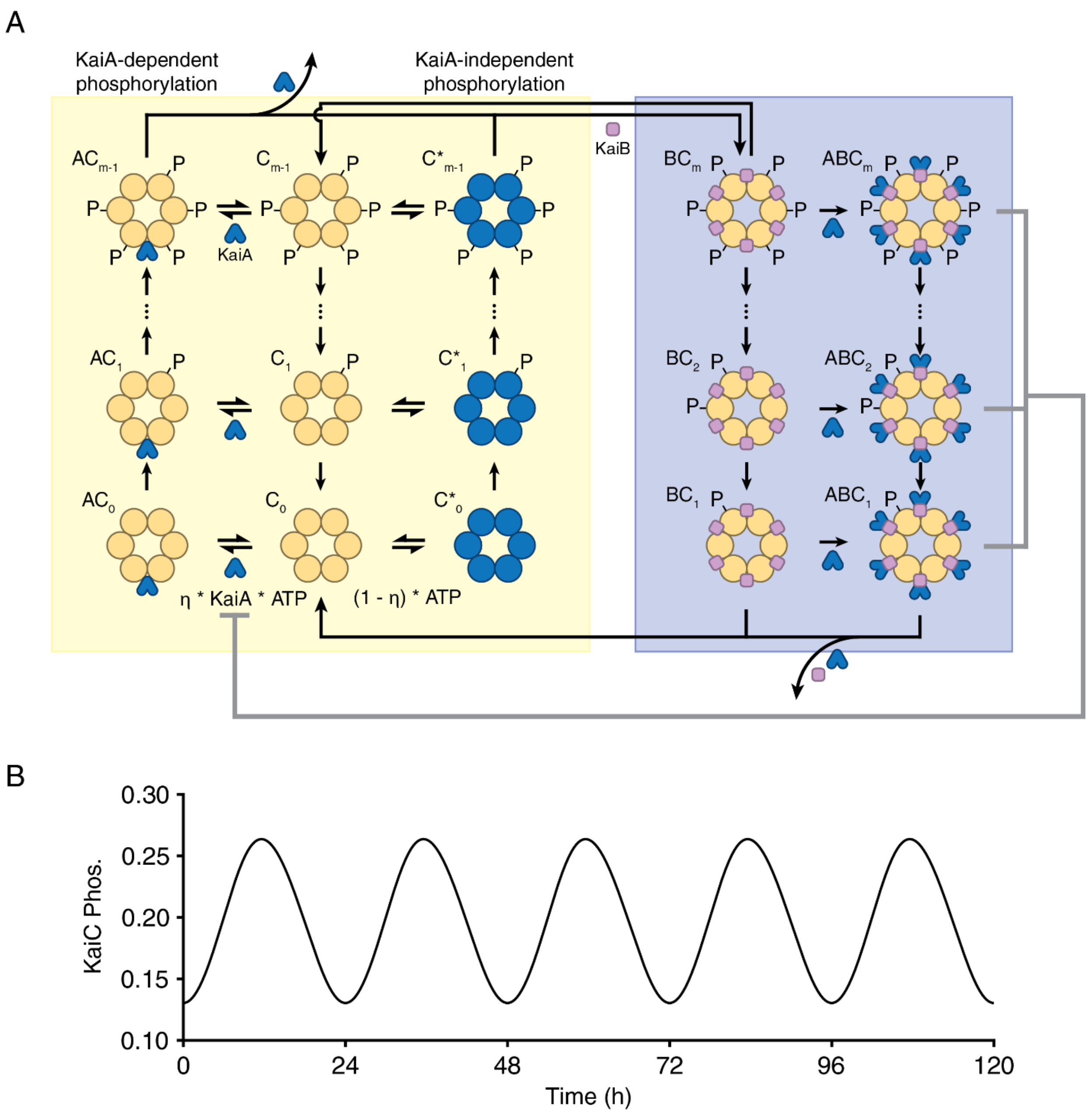

Supplementary Fig. 5

(**A**) Model of Kai system. KaiC is either in a phosphorylation-competent mode (*yellow box*) or a dephosphorylation mode (*blue box*), which corresponds to the ordered steps of phosphorylation and dephosphorylation observed experimentally. In the phosphorylation-competent mode, ground-state KaiC (*middle of yellow box*) can enter one of two states that allow for phosphorylation: KaiA-bound (*left*) and an equivalent state that is not KaiA-bound (*right*). These two states correspond to the scenario in which the KaiC C-terminal tails are exposed, promoting phosphorylation[19]. Feedback loop strength η determines the relative rates at which KaiC enters the KaiA-bound or KaiA-unbound phosphorylating state. KaiC undergoes a series of phosphorylation steps (a total of m steps, m = 6 shown here for illustrative purposes) until it becomes fully phosphorylated, unbinding KaiA if previously bound and binding to KaiB.

Dephosphorylating KaiC can either dephosphorylate as part of a KaiBC complex or it can do so as part of a ternary KaiABC complex which sequesters KaiA and forms the oscillator negative feedback loop. KaiC that becomes completely dephosphorylated unbinds KaiB and KaiA if previously bound, and the cycle restarts. (**B**) Deterministic simulation of model depicted in (A) with parameter m = 5 and numerically integrated with the fourth order Runge-Kutta method. The simulation was first initialized from 2.0 μM dephosphorylated KaiC hexamers and 2.0 μM KaiA dimers, and it was allowed to reach steady state oscillations by running the simulation for 1994.49 hours and saving the simulation state at that time. The simulation was then run for an additional 120 hours from the saved state, and the resulting trajectory is plotted here.

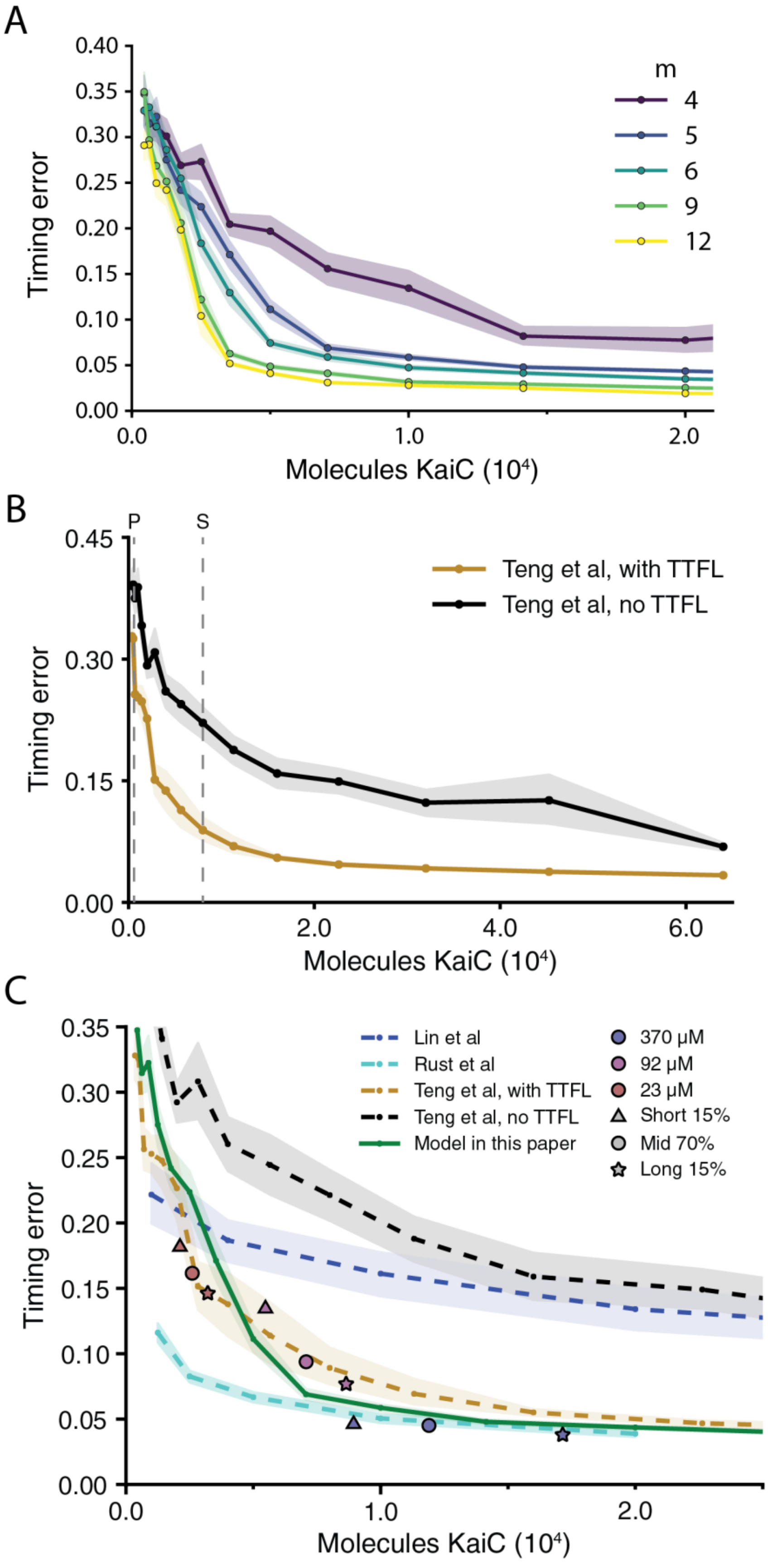

A
m
4
5
6
9
12
Timing error
Molecules KaiC (10⁴)
B
P
S
Teng et al, with TTFL
Teng et al, no TTFL
Timing error
Molecules KaiC (10⁴)
C
Lin et al
Rust et al
Teng et al, with TTFL
Teng et al, no TTFL
Model in this paper
370 μM
92 μM
23 μM
Short 15%
Mid 70%
Long 15%
Timing error
Molecules KaiC (10⁴)

Supplementary Fig. 6

(**A**) Different values of *m* (number of phosphorylations on KaiC required to switch from phosphorylating to dephosphorylating) show different noise scaling properties. Shown above is Kai copy number plotted against the coefficient of variation for various values of *m*. Shaded areas indicate 95% confidence intervals from bootstrapping (5000 iterations). The number of peak-to-peak intervals in each data point in each curve ranges from n = ~800 to n = ~1400. (**B**) Using a stochastic implementation of the model described in Teng et al[2], timing error per cycle was calculated in the presence (*brown*) or absence (*black*) of clock feedback on KaiC expression. This timing error is plotted as a function of the number of Kai molecules in the system in which the relative stoichiometry of all Kai species is preserved while the total copy number is varied. Vertical dashed lines indicate the wildtype KaiC copy number in *Prochlorococcus* (P) or *Synechococcus* (S). The shaded intervals around each curve indicate 95% confidence intervals from bootstrapping. (**C**) Shown are stochastic implementations of various models[2,6,8] and their timing errors as a function of Kai copy number. Shaded areas indicate 95% confidence intervals from bootstrapping. Triangles, circles, and stars indicate experimental data as shown in Figures 2D and 3D with error bars removed here for clarity.

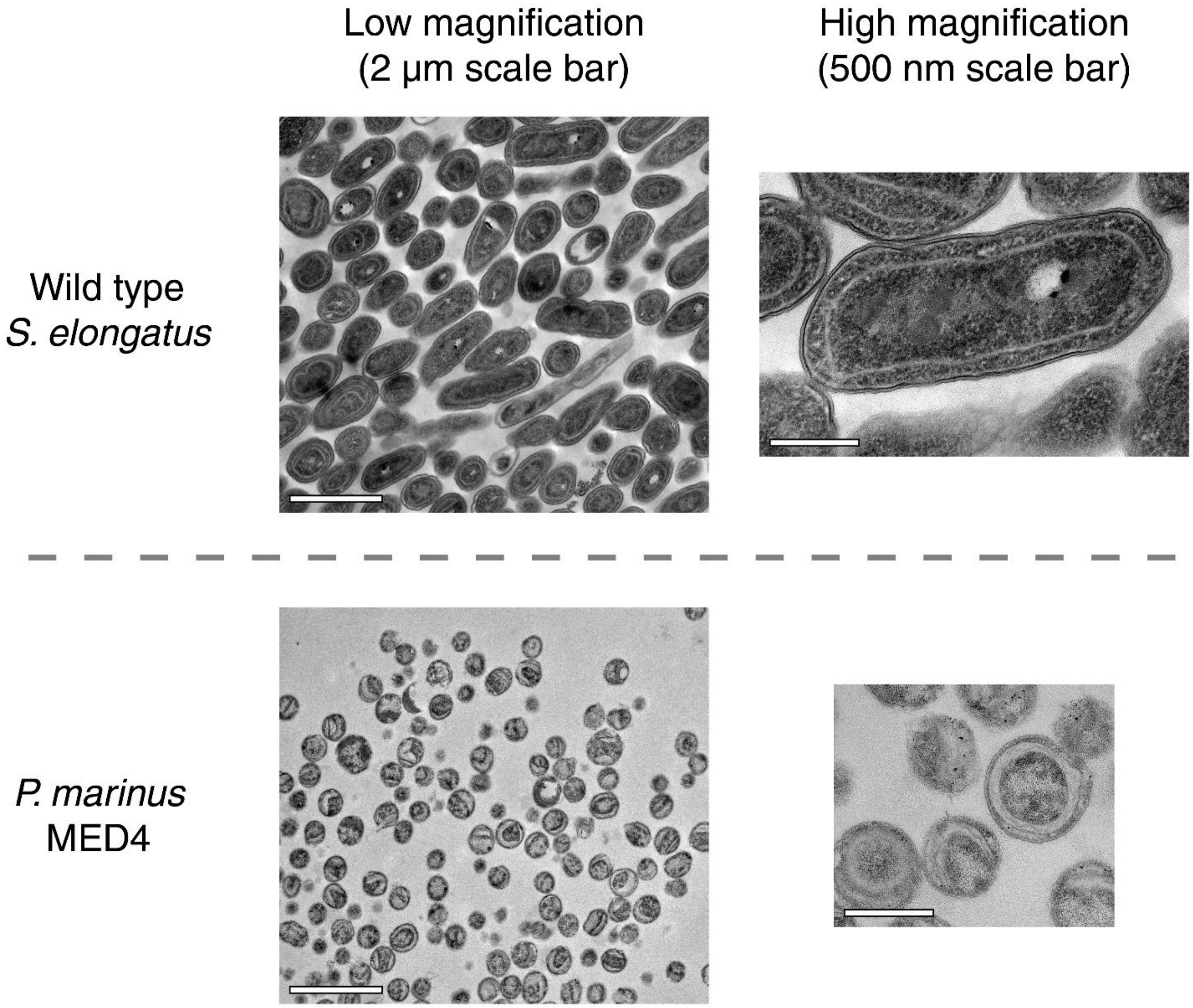

Supplementary Fig. 7

Electron micrographs of *S. elongatus* (*top*) and *P. marinus* MED4 (*bottom*). Two different magnifications are shown: low magnification (*left column*, scale bar: 2 um), and high magnification (*right column*, scale bar: 500 nm).

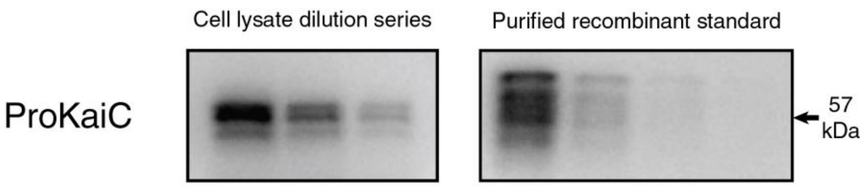

Supplementary Fig. 8

Representative western blot image of *Prochlorococcus* KaiC used for quantifying the cellular copy number (n = 3 biological replicates). *Left*: cell lysate from *Prochlorococcus* cells were loaded in a dilution series with 7.5 µg, 3.8 µg, and 1.9 µg of total protein loaded in each well from left to right. *Right*: purified recombinant standard of *Prochlorococcus* KaiC with 29 ng, 15 ng, 7.1 ng, and 3.6 ng loaded in each well from left to right.

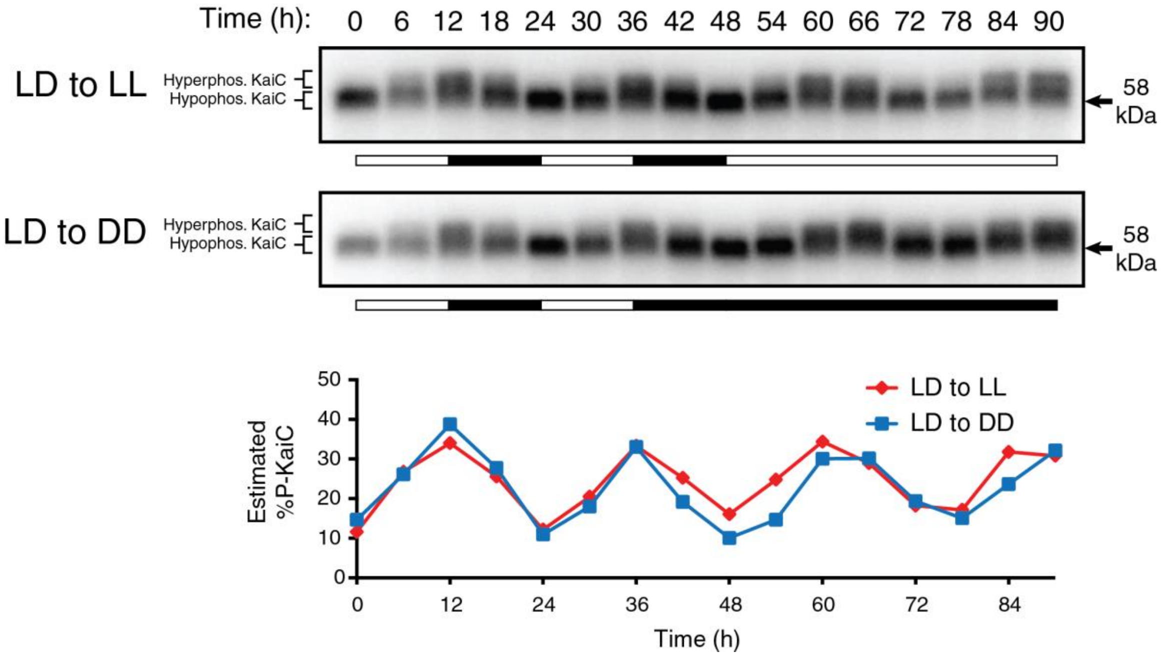

Supplementary Fig. 9

Top: western blot time course showing *Synechococcus* KaiC phosphorylation in cultures incubated in light-dark cycles followed by either constant light or constant dark. Due to ambiguity in band resolution, densitometry here only quantifies “hyperphosphorylated” vs “hypophosphorylated” KaiC. *Bottom*: quantification of estimated KaiC phosphorylation over time by densitometry.

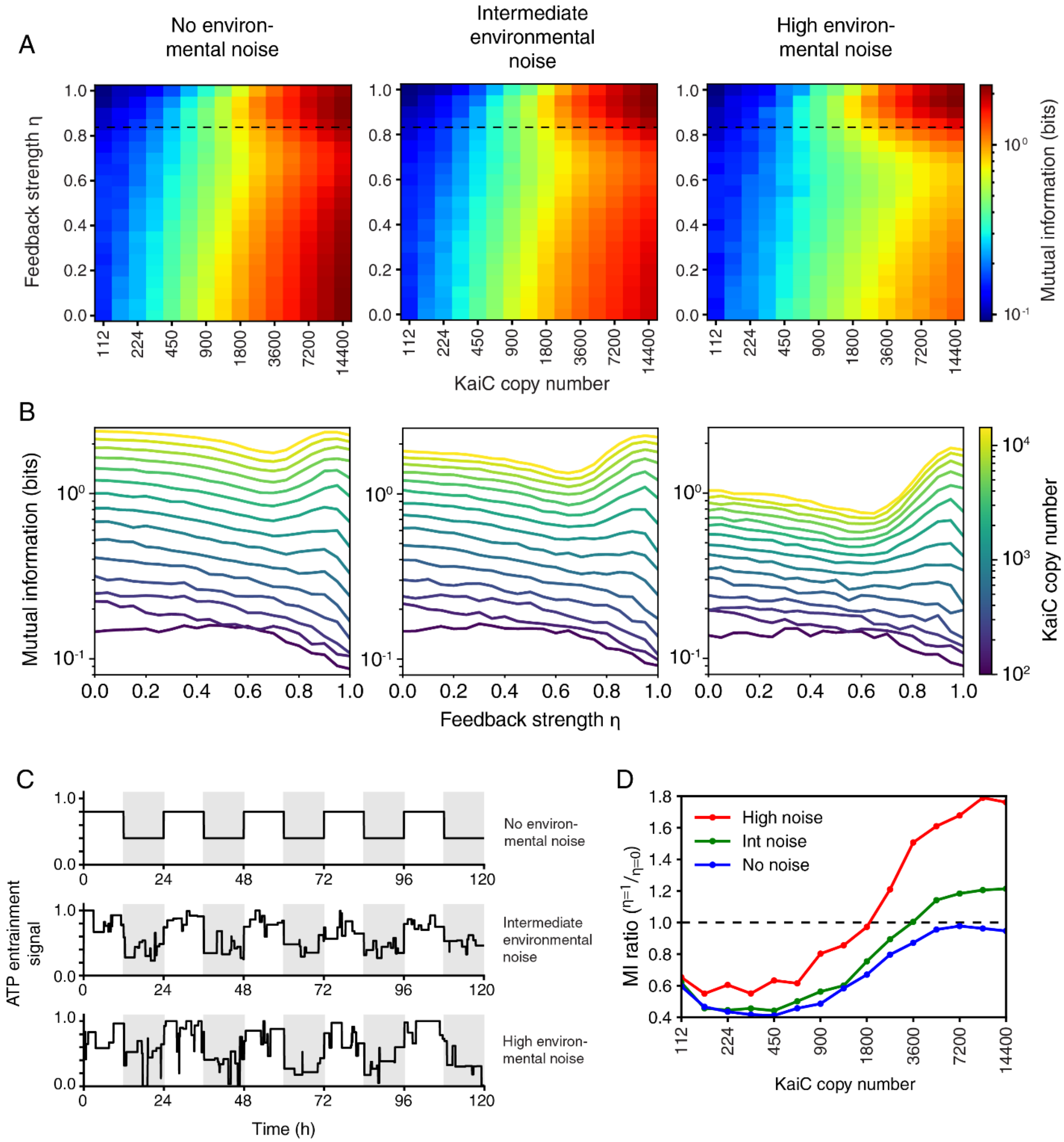

Supplementary Fig. 10

(**A**) Mutual information between the clock and time of day during light-dark cycles in the presence of environmental fluctuations of varying strength (fluctuation standard deviation σ = 0.1 for intermediate levels and 0.25 for high levels, see Supplementary Methods). Stable oscillations occur for feedback strength above 0.83 (*dashed line*). (**B**) Clock/time of day mutual information from model simulations at varying feedback strength and Kai copy number for varying amounts of environmental noise. Each curve corresponds to a single column in each heatmap in (A) and Fig. 4F, and the feedback strengths that maximize the mutual information of each curve corresponds to the points plotted in Fig. 4G. (**C**) ATP ratio environmental input with

no environmental fluctuations (*top*), an intermediate degree of fluctuations (*middle*), or a high degree of fluctuations (*bottom*). Alternating light and shaded backgrounds indicate the 12-hour day-night cycle. (**D**) Ratio of mutual information from an oscillator with a full strength feedback loop (η=1) over one with no feedback (η=0) with various levels of environmental fluctuations. The dashed line indicates the boundary at which systems with or without feedback have equal clock-environment mutual information.

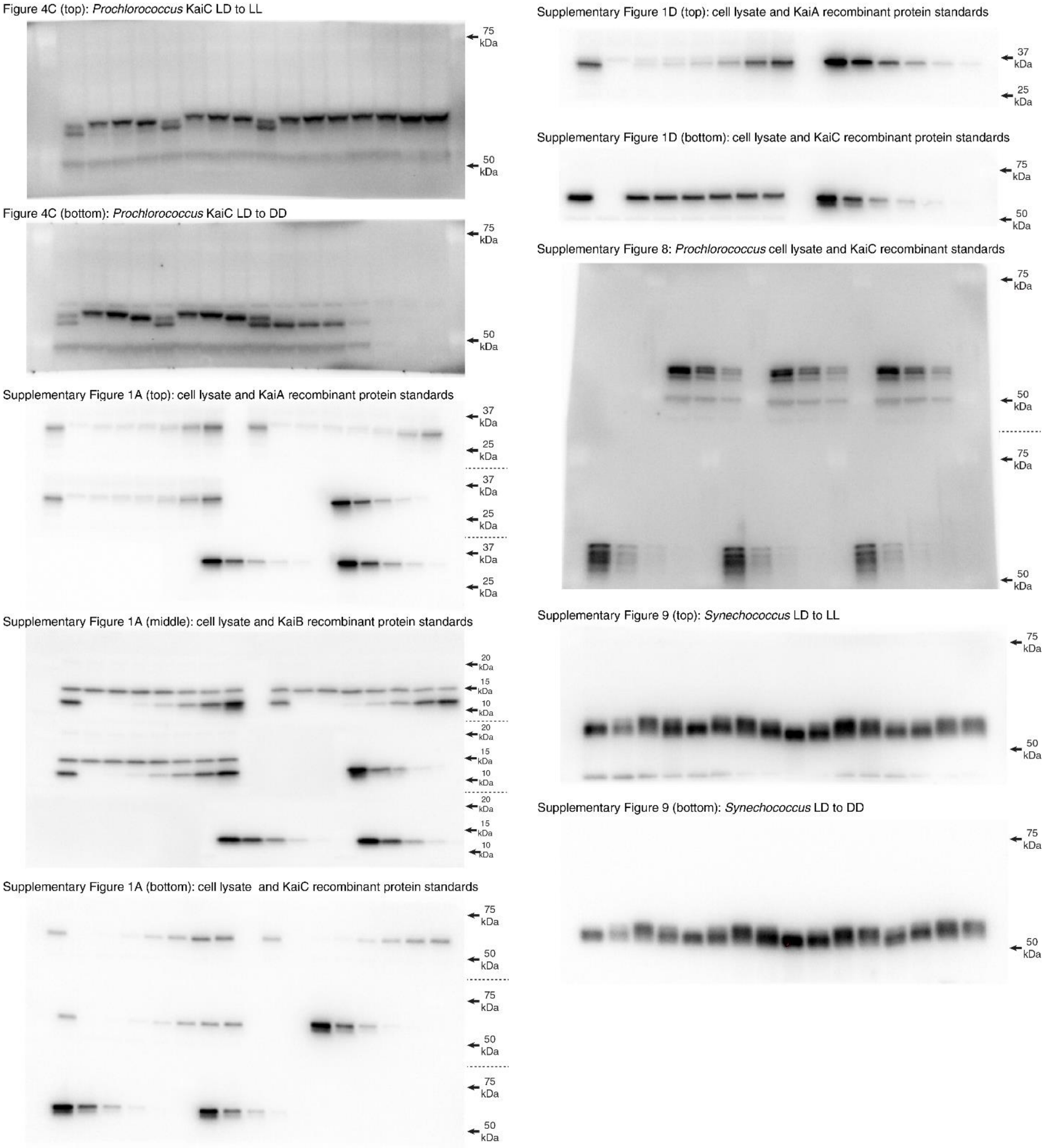

Supplementary Fig. 11

Raw, unprocessed western blot images corresponding to all western blots presented in this manuscript. Protein ladder molecular weights are indicated on each blot, and dotted lines denote divisions between different sections of SDS-PAGE gel that were transferred simultaneously onto the same, continuous membrane.

Supplementary References

1 Ma, A. T., Schmidt, C. M. & Golden, J. W. Regulation of gene expression in diverse cyanobacterial species by using theophylline-responsive riboswitches. *Applied and environmental microbiology* **80**, 6704-6713, doi:10.1128/aem.01697-14 (2014).

2 Teng, S. W., Mukherji, S., Moffitt, J. R., de Buyl, S. & O'Shea, E. K. Robust circadian oscillations in growing cyanobacteria require transcriptional feedback. *Science* **340**, 737-740, doi:10.1126/science.1230996 (2013).

3 Ito, H. *et al.* Cyanobacterial daily life with Kai-based circadian and diurnal genome-wide transcriptional control in Synechococcus elongatus. *Proc Natl Acad Sci U S A* **106**, 14168-14173, doi:10.1073/pnas.0902587106 (2009).

4 Vijayan, V., Jain, I. H. & O'Shea, E. K. A high resolution map of a cyanobacterial transcriptome. *Genome Biol* **12**, R47, doi:10.1186/gb-2011-12-5-r47 (2011).

5 Carpenter, A. E. *et al.* CellProfiler: image analysis software for identifying and quantifying cell phenotypes. *Genome Biol* **7**, R100, doi:10.1186/gb-2006-7-10-r100 (2006).

6 Lin, J., Chew, J., Chockanathan, U. & Rust, M. J. Mixtures of opposing phosphorylations within hexamers precisely time feedback in the cyanobacterial circadian clock. *Proc Natl Acad Sci U S A* **111**, E3937-3945, doi:10.1073/pnas.1408692111 (2014).

7 van Zon, J. S., Lubensky, D. K., Altena, P. R. & ten Wolde, P. R. An allosteric model of circadian KaiC phosphorylation. *Proc Natl Acad Sci U S A* **104**, 7420-7425, doi:10.1073/pnas.0608665104 (2007).

8 Rust, M. J., Markson, J. S., Lane, W. S., Fisher, D. S. & O'Shea, E. K. Ordered phosphorylation governs oscillation of a three-protein circadian clock. *Science* **318**, 809-812, doi:10.1126/science.1148596 (2007).

9 Tseng, R. *et al.* Structural basis of the day-night transition in a bacterial circadian clock. *Science* **355**, 1174-1180, doi:10.1126/science.aag2516 (2017).

10 Snijder, J. *et al.* Structures of the cyanobacterial circadian oscillator frozen in a fully assembled state. *Science* **355**, 1181-1184, doi:10.1126/science.aag3218 (2017).

11 Axmann, I. M. *et al.* Biochemical evidence for a timing mechanism in prochlorococcus. *J Bacteriol* **191**, 5342-5347, doi:10.1128/JB.00419-09 (2009).

12 Rust, M. J., Golden, S. S. & O'Shea, E. K. Light-driven changes in energy metabolism directly entrain the cyanobacterial circadian oscillator. *Science* **331**, 220-223, doi:10.1126/science.1197243 (2011).

13 Gillespie, D. T. Exact stochastic simulation of coupled chemical reactions. *The journal of physical chemistry* **81**, 2340-2361 (1977).

14 Qin, X. *et al.* Intermolecular associations determine the dynamics of the circadian KaiABC oscillator. *Proc Natl Acad Sci U S A* **107**, 14805-14810, doi:10.1073/pnas.1002119107 (2010).

15 Hayashi, F. *et al.* Stoichiometric interactions between cyanobacterial clock proteins KaiA and KaiC. *Biochem Biophys Res Commun* **316**, 195-202, doi:10.1016/j.bbrc.2004.02.034 (2004).

16 Dufresne, A. *et al.* Genome sequence of the cyanobacterium Prochlorococcus marinus SS120, a nearly minimal oxyphototrophic genome. *Proc Natl Acad Sci U S A* **100**, 10020-10025, doi:10.1073/pnas.1733211100 (2003).

17 Giovannoni, S. & Stingl, U. The importance of culturing bacterioplankton in the 'omics' age. *Nat Rev Microbiol* **5**, 820-826, doi:10.1038/nrmicro1752 (2007).
18 Brocchieri, L. & Karlin, S. Protein length in eukaryotic and prokaryotic proteomes. *Nucleic Acids Res* **33**, 3390-3400, doi:10.1093/nar/gki615 (2005).
19 Kim, Y. I., Dong, G., Carruthers, C. W., Jr., Golden, S. S. & LiWang, A. The day/night switch in KaiC, a central oscillator component of the circadian clock of cyanobacteria. *Proc Natl Acad Sci U S A* **105**, 12825-12830, doi:10.1073/pnas.0800526105 (2008).
