# Supplementary material for: High protein copy number is required to suppress stochasticity in the cyanobacterial circadian clock
Source: Nat Commun. 2018 Aug 1;9:3004. doi: 10.1038/s41467-018-05109-4 (PMC6070526; doi:10.1038/s41467-018-05109-4)
Supplement: Supplementary file 3 — Description of Additional Supplementary Files [file 41467_2018_5109_MOESM3_ESM.pdf]

Description of Additional Supplementary Files

File Name: Supplementary Movie 1
Description: Fluorescence microscope time-lapse movie showing wild type cells expressing EYFP-SsrA from the circadian *kaiBC* promoter. Scale bar 5 µm.

File Name: Supplementary Movie 2
Description: Time-lapse movie showing the copy number tunable strain incubated in 1 µM IPTG and 370 µM theophylline. Scale bar 5 µm.

File Name: Supplementary Movie 3
Description: Time-lapse movie showing the copy number tunable strain incubated in 1 µM IPTG and 92 µM theophylline. Scale bar 5 µm.

File Name: Supplementary Movie 4
Description: Time-lapse movie showing the copy number tunable strain incubated in 1 µM IPTG and 23 µM theophylline. Scale bar 5 µm.

File Name: Supplementary Data 1
Description: Kai protein copy number calculations.
